# The Lepidoptera of White Sands National Monument, Otero County, New Mexico, USA 4. A new species of *Schinia* Hübner, 1818 (Lepidoptera, Noctuidae, Heliothinae)

**DOI:** 10.3897/zookeys.149.1518

**Published:** 2011-11-24

**Authors:** Eric H. Metzler, Gregory S. Forbes

**Affiliations:** 1 Adjunct Curator of Lepidoptera, Michigan State University, P.O. Box 45, Alamogordo, NM 88311-0045 USA; 2 1009 Luna St., Las Cruces, NM 88003 USA

**Keywords:** Lepidoptera, Noctuidae, White Sands National Monument, Tularosa Basin, New Mexico, biological diversity, white gypsum dunes, National Monument, Otero County

## Abstract

In 2006 the U.S. National Park Service initiated a long term study of the Lepidoptera at White Sands National Monument, Otero County, New Mexico. *Schinia poguei* **sp. n.**, described here, was discovered in 2007, the second year of the study. The male and female adult moths and genitalia are illustrated.

## Introduction

The North American species of the genus *Schinia* Hübner, 1818 were revised by Hardwick (1996). In 2007, 2008, 2009, and 2010 adults of an undescribed species of *Schinia* Hübner were collected at White Sands National Monument, New Mexico. No specimens of this species were known prior to this study of insects at the Monument. The lack of specimens can probably be attributed to the dearth of insect collecting in the gypsum dunes ecosystem in New Mexico which is under the control of the U.S. National Park Service and the U.S. Army’s White Sands Missile Range.

## Methods

More than 250 samples of moths and other night flying insects were collected in U.S.D.A. type black light traps, and at black light, sometimes with mercury vapor light, and sheet, as described in Covell (1984), on 75 different nights. A detailed description of the study methods is in Metzler et al. (2009). Genitalia were examined following procedures outlined in Metzler and Forbes (2011a). Terminology for elements of wing pattern, morphology, and genital structures follow Forbes (1954), Hardwick (1970), (Lafontaine (1987, 2004), and Mikkola et al. (2009).

All specimens were collected as part of a long-term study of Lepidoptera at White Sands National Monument. Specimens are deposited in the following collections:

**EHM** Eric H. Metzler, Alamogordo, NM, for subsequent transfer to MSU

**MSU** Albert J. Cook Arthropod Research Collection, Department of Entomology, Michigan State University, East Lansing, MI

**NMSU** New Mexico State University Arthropod Collection, Las Cruces, NM

**UFL** McGuire Center for Lepidoptera and Biodiversity, University of Florida, Gainesville, FL

**UNM** Museum of Southwestern Biology, University of New Mexico, Albuquerque, NM

**USNM** provisionally deposited in United States Museum of Natural History (Smithsonian Institution), Washington, DC pending mutual resolution and agreement with the National Park Service regarding specimen deposition

**WHSA** White Sands National Monument, NM

## Results

### Schinia poguei

Metzler & Forbes sp. n.

urn:lsid:zoobank.org:act:D14AE8E8-6FD4-4B1B-A822-FBCE07620690

http://species-id.net/wiki/Schinia_poguei

**Figures 1–6.**
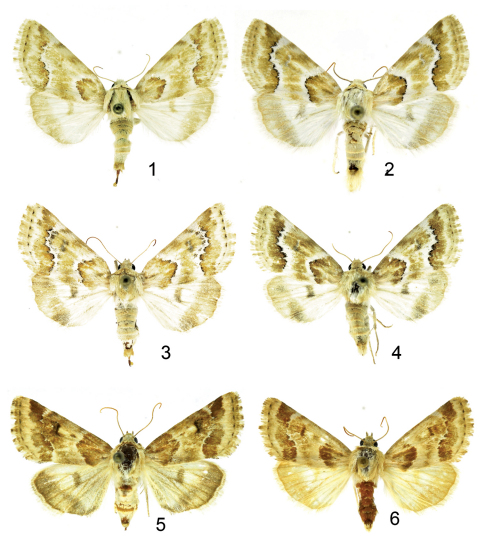
***Schinia***adults. **1** *Schinia poguei* Metzler & Forbes, female holotype **2** *Schinia poguei* Metzler & Forbes, male paratype **3** *Schinia poguei* Metzler & Forbes, female paratype **4** *Schinia poguei* Metzler & Forbes, male paratype **5** *Schinia walsinghami* Grote, female **6** *Schinia walsinghami* Grote, male.

Figs 1–4 7, 8, 11 13 14

#### Type material.

 Holotype female, pinned with label as follows: “USA: NM: Otero Co. White Sands Nat[ional] Mon[ument], interdune habitat, 106°10.84'W, 32°46.64'N, 4,008', 10 Oct[ober]. 2010, WsnmF, Eric H. Metzler, uv tr[a]p, Accss #: WHSA - 00131" “HOLOTYPE USNM *Schinia poguei* Metzler & Forbes” [red handwritten label] (USNM). Paratypes: 290 males and 107 females: NM: Otero Co. White Sands Nat[ional] Mon[ument] (hereafter WSNM) same data as Holotype. USA: NM: Otero Co. White Sands Nat[ional] Mon[ument], interdune habitat, 106°10.84'W, 32°46.64'N, 4,008', 4 Oct[ober]. 2010, WsnmF, Eric H. Metzler, uv tr[a]p, Accss #: WHSA - 00131 USA: NM: Otero Co. WSNM, interdune habitat, 106°11.49'W, 32°45.60'N, 4,000', 14 Sept[ember] 2009, WSNMB, Eric H. Metzler, uv tr[a]p, Accss #: WHSA - 00131. USA: NM: Otero Co. WSNM, interdune habitat, 106°11.38'W, 32°46.69'N, 4,000', 14 Sept[ember] 2009, WSNM8 Eric H. Metzler, uv tr[a]p, Accss #: WHSA - 00131. USA: NM: Otero Co. WSNM, interdune habitat, 106°11.32'W, 32°45.72'N, 4,000', 14 Sept[ember] 2009, WSNM9 Eric H. Metzler, uv tr[a]p, Accss #: WHSA - 00131. USA: NM: Otero Co. WSNM, 4000 ft. 32°45'41.4"N, 106°11'21.3"W, 5 X 2007, G. Forbes, 15w blacklight, interdune area with cottonwoods 2 mi SW Administration Bldg. ACCESSION NUMBER WHSA - 00131. USA: NM: Otero Co. WSNM, 4008 ft. Storage area W of Big Pedestal Rd. 23 IX 2008, G. Forbes, 15w blacklight, 32°46'42.3"N, 106°10'50.9"W, interdune scrub, ACCESSION NUMBER WHSA - 00131. USA: NM: Otero Co. WSNM, 4000 ft. 32°45'42.43"N, 106°11'18.57"W, 3 X 2008, G. Forbes, dune edge 50 m N of end Big Pedestal Rd. behind cottonwoods along road, ACCESSION NUMBER WHSA - 00131. USA: NM: Otero Co. WSNM, 4000 ft. 32°45'36.47"N, 106°11'28.22"W, interdune with cottonwoods 2 mi SW Admin Bldg, 15 bl[ac]kl[igh]t, 20 IX 2008, G. Forbes, ACCESSION NUMBER WHSA - 00131. USA: NM: Otero Co. WSNM, 4002 ft., Garton Pond, 2 mi. SE Vis. Ctr., 15 w blklight, 32°46'30.27"N, 106°08'42.96"W, 3 X 2008, G. Forbes, saltbush scrub, ACCESSION NUMBER WHSA - 00131. USA: NM: Otero Co. WSNM, 4008 ft., Storage area W of Big Pedestal Rd. 1 X 2008, G. Forbes, 15 w blacklight, 32°46'41.3"N, 106°10'50.9"W, interdune scrub, ACCESSION NUMBER WHSA - 00131. USA: NM: Otero Co. WSNM, 4008 ft., Storage area W of Big Pedestal Rd. 20 IX 2008, G. Forbes, 15 w blacklight, 32°46'41.3"N, 106°10'50.9"W, interdune scrub, ACCESSION NUMBER WHSA - 00131. USA: NM: Otero Co. WSNM, 3999 ft., 32°46'46.60"N, 106°10'26.70"W, 05 X 2007, G. Forbes, UV/MV lights, Admin. Bldg. Gypsum soil, Atriplex scrub, ACSN# WHSA - 00131. USA: NM: Otero Co. WSNM 3999 ft. 32°46'46.60"N, 106°10'26.70"W, 1 X 2008 G. Forbes 15w blacklight Admin bldg. Gypsum soil, Atriplex scrub Acsn# WHSA-00131. USA: NM: Otero Co. WSNM 4003 ft. 32°46'41.3"N, 106°10'50.9"W, 19 IX 2009 G. Forbes Boneyard storage area, Interdune scrub 15w blacklight trap Acsn# WHSA-00131. USA: NM: Otero Co. WSNM 4003 ft. 32°46'41.3"N, 106°10'50.9"W, 23 IX 2008 G. Forbes 15w blacklight, Boneyard storage area, Interdune Acsn# WHSA-00131. USA: NM: Otero Co. White Sands Nat[ional] Mon[ument], crest of dunes, 106°11.42'W, 32°45.69'N, 4,014', 4 Oct[ober]. 2010, WSNMC, Eric H. Metzler, uv tr[a]p, Accss #: WHSA - 00131. USA: NM: Otero Co. White Sands Nat[ional] Mon[ument], crest of dunes, 106°11.42'W, 32°45.69'N, 4,014', 10 Oct[ober]. 2010, WSNMC, Eric H. Metzler, uv tr[a]p, Accss #: WHSA - 00131. USA: NM: Otero Co. White Sands Nat[ional] Mon[ument], edge of dunes, 106°11.33'W, 32°45.70'N, 4,001', 4 Oct[ober]. 2010, WSNM3, Eric H. Metzler, uv tr[a]p, Accss #: WHSA - 00131. USA: NM: Otero Co. White Sands Nat[ional] Mon[ument], edge of dunes, 106°11.33'W, 32°45.70'N, 4,001', 10 Oct[ober]. 2010, WSNM3, Eric H. Metzler, uv tr[a]p, Accss #: WHSA - 00131. USA: NM: Otero Co. White Sands Nat[ional] Mon[ument], interdune vegetation, 106°10.82'W, 32°46.62'N, 4,008', 4 Oct[ober]. 2010, WSNMD, Eric H. Metzler, uv tr[a]p, Accss #: WHSA - 00131 Paratypes are deposited with NMSU, UNM, MSU, EHM, UFL, USNM, and WHSA.

**Figures 7–12.**
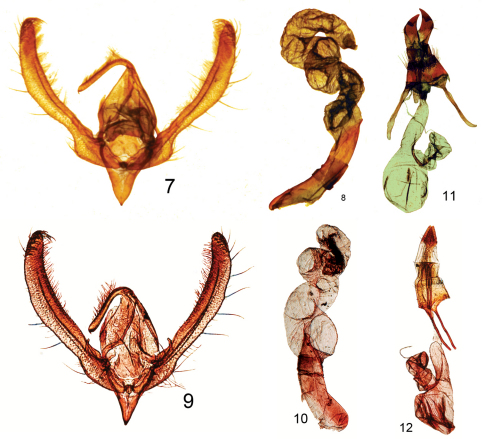
**7–10 *Schinia***male genitalia. **7** *Schinia poguei* Metzler & Forbes, male genitalia paratype **8** *Schinia poguei* Metzler & Forbes, male aedeagus paratype **9** *Schinia walsinghami* Grote, male genitalia **10** *Schinia walsinghami* Grote, male genitalia aedeagus; **11–12** *Schinia* female genitalia **11** *Schinia poguei* Metzler & Forbes, female genitalia paratype **12** *Schinia walsinghami* Grote, female genitalia**.**

**Figure 13.**
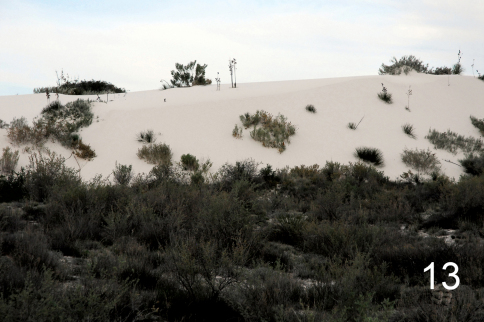
White dune habitat of type locality of *Schinia poguei*.

**Figure 14.**
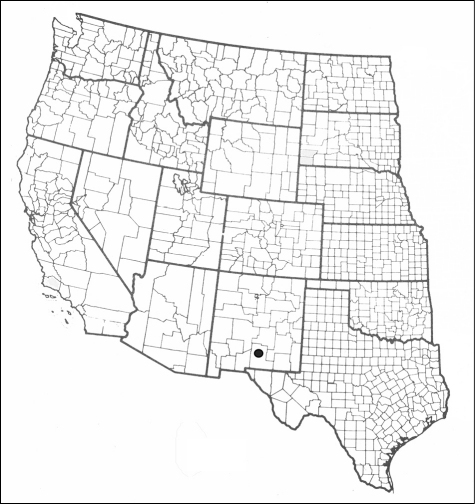
Distribution map for *Schinia poguei*.

#### Etymology.

 The specific epithet of this species, *poguei*, a noun in the genitive case, recognizes Michael G. Pogue’s contributions to the study of Lepidoptera. Mike Pogue and Metzler share a personal friendship going back to the early 1990s. We are pleased to name this species for Mike Pogue.

#### Diagnosis.

*Schinia poguei* (figs 1–4), a pale tan moth with white markings and darker accents, looks most like pale specimens of *Schinia walsinghami* (Henry Edwards, 1881) (figs 5, 6, 9, 10, 12). The diagnostic features are in the genitalia and the color of the maculation. In the female genitalia: 1) the papilla anales (ovipositor lobes) of *Schinia poguei* are curved upward, and the papilla anales of *Schinia walsinghami* are V shaped and not curved; 2) on the 9^th^ abdominal segment the minute spiculation is less dense in *Schinia poguei*, and the spicules are wider in *Schinia poguei* than in *Schinia walsinghami*; 3) on the 8th segment the setae are long and dense in *Schinia poguei* and shorter and more sparse in *Schinia walsinghami*; and 4) the anterior and posterior apophyses are thicker in *Schinia poguei* than in *Schinia walsinghami*. In the male genitalia the ampulla of *Schinia poguei* is short and broad, and in *Schinia walsinghami* the ampulla is twice as long and one half as wide as *Schinia poguei*. At White Sands National Monument, where the two species are sympatric, *Schinia walsinghami*, which flies in August and September, is a gray-brown moth with white markings, whereas *Schinia poguei* flies in September and October and is a pale tan moth with white markings.

#### Description.

**Adult male** (figs 2, 4): *Head:* front closely scaled, pale tan; vertex scales narrow, strap-like, rough, pale tan. Labial palpus pale tan; erect, scales hair-like and strap-like, closely scaled laterally and mesally, longer scales form longer ragged fringe ventrally and shorter fringe dorsally. Haustellum coiled between labial palpi with three complete loops. Antenna filiform, dorsally pale tan, closely scaled, ventrally naked, brown. *Thorax:* dorsum pale tan, scales long hair-like or strap-like; underside whitish, scales erect long hair-like. Legs: coxa and femur whitish, closely scaled with long hair-like scales on ventral surface forming a shaggy fringe; fore tibia pale tan, closely scaled, spine-like setae, stout on lateral margins, apex with one inner and one outer long stout spine-like seta; mid and hind tibia, tibial spines, and tarsomeres pale tan, closely scaled. Fore wing: Length 9.0–13.0 mm, mean 11.5 mm, n = 72, pale tan; antemedial line strongly excurved, white, lined with black and/or dark-brown scales basally; postmedial line strongly excurved over cell and recurved to inner margin, white, lined with black and/or dark-brown scales distally, vague basally; subterminal line a white shade; terminal line a series of small black spots; orbicular spot obscure; reniform spot crescent shaped, white, sometimes highlighted with a smudge of darker scales; fringe pale tan; underside whitish, upperside markings vaguely visible; fringe whitish. Hind wing: whitish, outer one-third from tornus to inner margin ranges from fuscous to pale tan, discal spot obscure; fringe whitish; underside white. *Abdomen:* dorsum closely scaled, pale tan; underside whitish, closely scaled. Genitalia (figs 7, 8): Tegumen expanded laterally, lateral lobes at junctures with valvae, narrowed at dorsum; uncus cylindrical, apex short, acutely pointed, turned downward, length = 0.34 × length of valve; saccus short, V shaped, apex acute; juxta shield shaped, slightly cleft on anterior margin; valve elongate, narrow, width of valve at widest part = 0.12 × length of valve, slightly curved, sacculus well developed, ampulla well developed, short and broad, length = 0.03 × length of valve; cucullus not well differentiated, corona with 16–18 mesally directed curved strong setae. Aedeagus with sclerotized process directed anteriorly at approximate mid point, vesica with 3 coils, flattened subbasal diverticulum present. **Adult female** (figs 1, 3): similar to male. Forewing length 10.5–13.0 mm, mean 12.1 mm, n = 46. Genitalia (fig. 11): Papilla analis sclerotized, flattened, curved upward, broad at base, apex pointed, not fused; posterior apophysis flattened, widened, extending anteriorly to slightly beyond anterior margin of 8^th^ abdominal segment; anterior apophysis flattened, widened, length similar to posterior apophysis; 8^th^ abdominal segment ringed with elongate setae; ductus bursa lightly sclerotized near posterior end, elongate; corpus bursa ovate with 4 elongate signa; appendix bursae with 3 coils.

#### Remarks.

 This new species is placed in the genus *Schinia* Hübner, 1818, based on the appearance of the imago and structure of the male and female genitalia. The spiculation on the 9^th^ abdominal segment is best seen with a compound microscope. A female was selected as holotype because the differences in the papilla analis between *Schinia walsinghami* and *Schinia poguei* can be seen in situ without dissection. Individual specimens of *Schinia poguei* may be lighter or darker in color. Darker specimens appear to have more complete forewing patterns on upper and undersides. Abdomens tend to grease, and sometimes the grease invades the wings.

#### Distribution and biology.

*Schinia poguei* occurs in White Sands National Monument, Otero County, New Mexico (figs 13, 14). Adults were collected in black light traps and at black light, or mercury vapor light, and sheet placed within or adjacent to the white gypsum dunes, and interdunal areas. The immature stages are unknown.

#### Discussion.

In 2006 the U.S. National Park Service invited Metzler to initiate a long-term study of the Lepidoptera at White Sands National Monument, Otero County, New Mexico. A primary purpose of the study was to compile an inventory of moths in habitats within and immediately adjacent to the white gypsum dunes in the Monument.

White Sands National Monument preserves 284.9 km^2^ (110 square miles), or about 40%, of the world’s largest snow-white gypsum dune field. The remainder of the 275 square miles dune field is under the jurisdiction of the U.S. Army in the White Sands Missile Range. The dune field is located in the northern Chihuahuan Desert in southern New Mexico’s Tularosa Basin (Schneider-Hector 1993). In 1950 Stroud reported twenty species of Lepidoptera from the Monument. In the period 9 February 2007 through 31 December 2010 Metzler and Forbes identified more than 430 species of Lepidoptera (unpublished data) from the Monument. A complete description of the study site and some of its unique biological resources is in Metzler et al. (2009).

This is the sixth in a series of papers (Metzler et al. 2009, 2010; Metzler and Forbes 2011a, 2011b) pertinent to a detailed study of the Lepidoptera at White Sands National Monument. This is the fourth species of moth, described as part of this study, and the third species that appears to be a white species (Kain 2000). The study of Lepidoptera at White Sands National Monument by Metzler and Forbes is projected to last approximately 10 years.

## Supplementary Material

XML Treatment for Schinia poguei
